# Role of Osteopontin in Idiopathic Pulmonary Fibrosis

**DOI:** 10.1371/journal.pmed.0020314

**Published:** 2005-09-06

Idiopathic pulmonary fibrosis is a chronic progressive scarring disease of the lung in which gradually the walls of the air sacs of the lungs become replaced by fibrotic tissue. When scarring forms, there is an irreversible loss of the tissue's ability to transfer oxygen into the bloodstream.

The disease affects more than 5 million people worldwide, of whom 40,000 die every year. Misdiagnosis is common because the origin and development of the disease is not completely understood. There are also no effective treatments; drugs to treat lung scarring are still in the experimental phase, and treatments to suppress inflammation have no beneficial effect in most patients.

Although significant advances have been made in the characterization of the clinical features of this disease, the molecular mechanism in humans is still largely unknown. In general, pulmonary fibrosis might be the result of an autoimmune disorder, the after effects of a viral infection, or a genetic condition. Pulmonary fibrosis also occurs after inhalation of some pollutants, in association with diseases such as scleroderma, rheumatoid arthritis, lupus, and sarcoidosis, and after certain medications and therapeutic radiation. However, the etiology of idiopathic pulmonary fibrosis is presently unknown.

Now Annie Pardo and colleagues examine the role of osteopontin, which has diverse functions as a cell-adhesion and migration molecule, in the pathogenesis of idiopathic pulmonary fibrosis. Osteopontin is a multifunctional cytokine that has been implicated in several physiological and pathological processes including bone resorption, malignant transformation, and metastasis. It is also considered a key molecule for regulating inflammation, cellular immune response, and tissue repair, with a unique effect on T cell function. [Fig pmed-0020314-g001]

**Figure pmed-0020314-g001:**
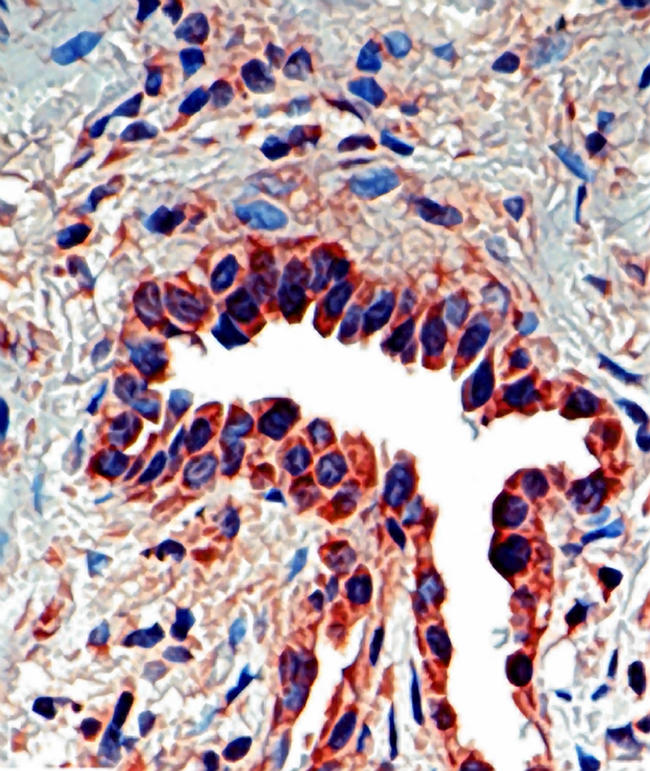
Osteopontin protein in lung from patient with IPF

Using oligonucleotide microarrays these researchers have previously demonstrated that osteopontin is highly upregulated in bleomycin-induced lung fibrosis in mice—an animal model of pulmonary fibrosis.

In the current study, they used microarrays to analyze gene expression patterns in lung samples (13 samples from people with idiopathic pulmonary fibrosis and 11 from control individuals). They found that osteopontin was the most upregulated gene in the lungs of patients with idiopathic pulmonary fibrosis, and that it was mainly expressed by alveolar epithelial cells.

To better understand the potential local profibrotic effects of osteopontin they then studied its effects on lung fibroblasts and alveolar epithelial cells and found that osteopontin induced a significant increase in migration and proliferation in both fibroblasts and epithelial cells. However, although the effect on fibroblast migration/proliferation was dependent mainly on integrins, in epithelial cells proliferation was mainly dependent on CD44 and migration was dependent on CD44 and integrin signaling.

Osteopontin also showed profibrotic-relevant effects on molecules involved in extracellular matrix remodeling. For example, in fibroblasts osteopontin increased TIMP-1 and type I collagen and inhibited MMP-1 expression, whereas in alveolar epithelial cells it induced MMP-7.

These findings concur with previous studies in experimental tissue fibrosis that have suggested a possible profibrotic role of osteopontin, said the authors. For example in kidney fibrosis, osteopontin enhances macrophage recruitment and stimulates the development of renal scarring after an acute ischemic insult; most importantly, mice that do not express the gene for osteopontin are protected from lung fibrosis induced by the drug bleomycin.

Altogether the results suggest a mechanism to explain most of the profibrotic effects of osteopontin by direct effects on fibroblasts and epithelial cells in the lungs. The findings also suggest that the interaction between MMP-7 and osteopontin might be involved in the progressive nature of the disease. Osteopontin is a potential target for therapeutic intervention in this relentless, incurable disease.

